# New Cortisol Thresholds for the Diagnosis of Adrenal Insufficiency Using the Low-Dose Synacthen Test in Children on Long-Term Corticosteroids: A North African Pilot Study

**DOI:** 10.3390/diagnostics16071065

**Published:** 2026-04-01

**Authors:** Taieb Ach, Abdelbari Marwa, Ben Hadj Ali Wiem, Marzouk Hajer, Wiem Saafi, Zarrouk Oumayma, Tej Amel, Kbaili Raoudha, Jaballah Nesrine, Bouguila Jihene, Soyah Najla, Hamza El Fekih, Saad Ghada, Debbabi Wided, Monia Zaouali, Yosra Hasni, Boughammoura Lamia

**Affiliations:** 1 Faculty of Medicine of Sousse, University of Sousse, Sousse 4003, Tunisia; marwa.abdelbari@famso.u-sousse.tn (A.M.); wiem2014@gmail.com (B.H.A.W.); wiem.saafi@famso.u-sousse.tn (W.S.); oumaymazarrouk59@gmail.com (Z.O.); raoudha.kbaili@famso.u-sousse.tn (K.R.); nesrine.jaballah@famso.u-sousse.tn (J.N.); jihene.bouguila@famso.u-sousse.tn (B.J.); najla.soyah@famso.u-sousse.tn (S.N.); hamza.elfekih@famso.u-sousse.tn (H.E.F.); ghada.saad@famso.u-sousse.tn (S.G.); yosra.hasni@famso.u-sousse.tn (Y.H.); lamia.boughammoura@famso.u-sousse.tn (B.L.); 2 Department of Endocrinology, University Hospital of Farhat Hached, Sousse 4000, Tunisia; 3 Laboratory of Exercise Physiology and Pathophysiology, L.R.19ES09, Sousse 4000, Tunisia; 4 Department of Pediatrics, Faculty of Medicine of Sousse, Sousse 4000, Tunisia; 5 Department of Endocrinology, University Hospital Ibn El Jazzar, Kairouan 3100, Tunisia; hajer.marzouk@famso.u-sousse.tn (M.H.); wided.debbabi@famso.u-sousse.tn (D.W.)

**Keywords:** synacthen, cortisol, cut-off, adrenal insufficiency

## Abstract

**Background:** The low-dose Synacthen stimulation test (LD-SST) is the reference dynamic test for diagnosing glucocorticoid-induced adrenal insufficiency (AI) in children, but it is resource-intensive and costly. This study aimed to establish morning cortisol thresholds predictive of hypothalamic–pituitary–adrenal (HPA) axis response to LD-SST in pediatric patients receiving chronic corticosteroid therapy. **Methods:** We conducted a prospective study including 71 children (mean age 6.23 ± 3.49 years; 57.7% male) receiving prolonged oral or inhaled corticosteroids. All patients underwent LD-SST with cortisol measurements at 0, 30, and 60 min. Patients were classified as having AI (peak cortisol < 18 μg/dL at T30 or T60, *n* = 39) or normal adrenal function (peak cortisol ≥ 18 μg/dL, *n* = 32). ROC curve analysis determined optimal cortisol thresholds for predicting AI. **Results:** Among the 71 patients, 44 received inhaled corticosteroids (62%) and 27 oral corticosteroids (38%). Asthma (61.9%) and autoimmune diseases (18.3%) were the main indications. The prevalence of AI was 54.9% (*n* = 39). Mean morning cortisol was significantly lower in the AI group compared to the normal group (6.69 ± 1.99 vs. 9.21 ± 2.49 μg/dL, *p* < 0.001). ROC analysis identified a morning cortisol <6 μg/dL as predictive of AI with 96.9% sensitivity and 46.2% specificity (AUC = 0.823), while a threshold >13 μg/dL predicted normal HPA function with 100% specificity. Using these thresholds would have avoided LD-SST in 29.6% of patients. The cortisol increment following stimulation also demonstrated diagnostic value (AUC = 0.822), with an increment <9 μg/dL suggesting AI and ≥9 μg/dL likely excluding the diagnosis. **Conclusions:** In this North African pilot study, morning cortisol measurement at 7 days after oral corticosteroid withdrawal predicted LD-SST response. A threshold of ≤6 μg/dL identified patients with persistent HPA suppression with high sensitivity (97.4%), while ≥13 μg/dL identified patients with preserved function with high specificity (100%). However, the 7-day washout period might be insufficient for complete HPA recovery; therefore, these thresholds reflect residual suppression rather than permanent adrenal insufficiency.

## 1. Introduction

Glucocorticoids (GCs) are considered a cornerstone of the therapeutic arsenal in pediatrics. Since their discovery in the 1940s, both systemic and inhaled forms of GCs have demonstrated efficacy across a wide range of conditions and are now routinely prescribed in pediatric practice [1]. Despite their therapeutic benefits, the burden of glucocorticoid-related adverse effects remains a significant clinical concern, prompting ongoing research into risk mitigation strategies [2].

Long-term GC therapy is particularly indicated in the management of asthma, as well as eczema, autoimmune diseases, and acute leukemia [3,4,5,6]. However, the use of GCs is associated with numerous adverse effects, which may occur early or be delayed. These complications can outweigh the therapeutic benefits of treatment, leading to significant morbidity, mortality, and impaired quality of life. GCs can suppress the hypothalamic–pituitary–adrenal (HPA) axis, resulting in adrenal insufficiency (AI). The onset of AI is often unpredictable and may represent a serious condition [7]. Iatrogenic suppression of the HPA axis has been reported in a wide range of patients, with recent meta-analyses estimating prevalence rates between 15 and 60% depending on dosage, duration, and route of administration [8,9].

The diagnosis of AI relies on dynamic testing of the HPA axis. The insulin tolerance test (ITT), also known as the insulin-induced hypoglycemia test, is considered the gold standard. However, it is rarely used in pediatrics and is contraindicated in infants under 2 years of age because of the risk of hypoglycemia-induced brain injury. Thus, the ACTH (synacthen) stimulation test is usually performed as a first-line investigation, especially in young infants, since circadian rhythm is not established until about 4 months of age [10]. Emerging evidence suggests that the low-dose (1 μg) Synacthen stimulation test (LD-SST) may have better sensitivity than the standard-dose test [11].

Nevertheless, dynamic testing can incur significant socioeconomic costs. To mitigate this, some researchers have proposed morning cortisol [12] or ACTH [13] thresholds as predictive markers of normal HPA function, potentially avoiding unnecessary stimulation tests. However, data in pediatric populations remain limited and highly heterogeneous. Most existing studies have been conducted in Western countries, and to our knowledge, no research has been carried out in Africa to define a region-specific cortisol threshold.

Given the life-threatening nature of adrenal crises, establishing accurate, accessible, and cost-effective diagnostic strategies for glucocorticoid-induced AI in children is an urgent priority. This study aims to establish a cortisol threshold predictive of a normal HPA axis response following the LD-SST in children, with the goal of improving identification of at-risk patients and preventing adrenal crises.

This study aims to establish a cortisol threshold predictive of a normal HPA axis response following the LD-SST in children.

## 2. Materials and Methods

### 2.1. Patients

This prospective, descriptive, and analytical study included 71 children receiving treatment for chronic conditions requiring prolonged corticosteroid therapy (either oral or inhaled) for more than three weeks.

Included patients were children aged 2–14 years receiving long-term corticosteroid therapy for >3 weeks were eligible. Exclusion criteria included: (1) inability to safely discontinue oral corticosteroids for one week due to severe underlying disease or history of adrenal crisis; (2) non-adherence to treatment; (3) parental refusal; (4) inadequate sample quality. Of 84 eligible patients, 13 were excluded: 8 due to inability to safely discontinue oral steroids (severe asthma *n* = 5, recent adrenal crisis *n* = 3), and 5 due to consent withdrawal.

The 7-day washout period was designed to eliminate exogenous steroid from the circulation to allow measurement of endogenous cortisol without pharmacological interference. However, this duration is insufficient for HPA axis recovery, which may require weeks to months depending on the duration and potency of prior corticosteroid exposure. Consequently, our results reflect the prevalence of adrenal suppression one week after steroid withdrawal rather than the prevalence during active treatment or after complete recovery.

Children receiving oral corticosteroids were administered therapeutic (supraphysiological) doses for treatment of their underlying disease, not replacement doses for adrenal insufficiency. The dose range of 12–15.5 mg/m^2^/day of hydrocortisone equivalent refers to the treatment doses used for their conditions.

The LD-SST was performed at the end of the prescribed treatment course, after patients had completed their indicated duration of corticosteroid therapy. This represents a post-treatment assessment to determine whether HPA axis recovery had occurred, allowing safe discharge from endocrine follow-up without need for stress dosing precautions.

Among those receiving inhaled corticosteroids (ICS), 39 patients (88.6%) were on medium doses, while 5 patients (11.4%) received high doses, according to the 2022 Cochrane review, which takes patient age into account [14]. For these patients, the LD-SST was performed during ongoing maintenance therapy to screen for glucocorticoid-induced adrenal insufficiency as part of routine monitoring.

The study was conducted from January 2023 to January 2024 in the Pediatrics and Endocrinology Departments of Farhat Hached Hospital in Sousse. Laboratory analyses were performed at the Hormonal Physiology Laboratory of the Faculty of Medicine of Sousse.

Patients were excluded if their biological samples did not meet the required delivery and storage standards, if parental consent was not obtained, or if they were considered non-adherent to their corticosteroid treatment.

For each included patient, the following data were recorded: age, sex, family and personal medical history, indication for corticosteroid therapy, duration, dosage, and the specific corticosteroid used. Clinical assessment comprised a comprehensive physical examination, anthropometric measurements, respiratory evaluation, blood pressure measurement, capillary blood glucose levels, and pubertal staging according to Tanner criteria.

Biological investigations included a complete blood count, calcium and phosphate panel, renal function tests, electrolyte balance, and thyroid function tests when available.

### 2.2. Evaluation of HPA Axis by Dynamic Tests

The LD-SST was performed under fasting conditions between 8:00 and 9:00 a.m. at the Pediatric Day Hospital of Farhat Hached Hospital.

For patients receiving oral corticosteroids: Treatment was discontinued one week before the LD-SST. This duration was selected based on pharmacokinetic principles, as five half-lives of prednisone/prednisolone is approximately 10–20 h, making one week sufficient for complete drug clearance. However, we emphasize that this one-week period allows assessment of endogenous HPA function in the absence of exogenous steroids, but does not imply complete HPA axis recovery, which may take months to years. During this washout period, patients were closely monitored for symptoms of adrenal insufficiency, and parents received comprehensive education regarding stress dosing and emergency procedures. High-risk patients (those with severe asthma, recent adrenal crisis, or very high corticosteroid doses) were not subjected to complete discontinuation; instead, their dose was tapered to physiological hydrocortisone replacement (8–10 mg/m^2^/day) during the week preceding testing. No patient experienced symptomatic adrenal insufficiency during this period.

For patients receiving inhaled corticosteroids: Treatment was continued at the usual dose until the morning of the test. This approach was chosen because: (a) inhaled corticosteroids have minimal systemic bioavailability, (b) discontinuation could precipitate asthma exacerbation, and (c) our objective was to assess HPA function under real-world treatment conditions. This methodology is consistent with recent pediatric studies evaluating inhaled corticosteroid effects on the HPA axis [14,15].

A 250 µg Synacthen ampoule was diluted to prepare 1 µg doses for intramuscular (IM) injection. Baseline blood samples were collected for cortisol and ACTH measurements at time 0 min (T0). Subsequently, 1 µg of Synacthen was administered via IM injection, and blood samples for cortisol were collected at 30 min (T30) and 60 min (T60) post-injection. Samples for cortisol measurement were collected in dry tubes, whereas those for ACTH were collected in heparinized tubes, with strict adherence to the cold chain for preservation. Throughout the test, any adverse effects were monitored by clinical examination and patient questioning, conducted concurrently with each blood draw.

The term “peak cortisol frequency” refers to the proportion of patients in whom the maximum (peak) cortisol response to Synacthen stimulation occurred at each time point (T30 vs. T60). This parameter provides insight into the temporal dynamics of the adrenal response to Synacthen stimulation. In individuals with normal HPA axis function, cortisol typically peaks earlier (at T30) and may decline by T60. In contrast, patients with adrenal insufficiency may show a delayed or blunted response, with peak cortisol occurring later (at T60) or failing to reach diagnostic thresholds altogether.

### 2.3. Assessment of Hormone Levels

Serum cortisol levels were measured using the radioimmunoassay (RIA) method with a kit method (Beckman Coulter, Brea, CA, USA) with a sensitivity of 7 ng/mL; the intra-assay and inter-assay coefficient variation was 2.8% and 5.3%, respectively.

### 2.4. Distribution of Groups

- Group 1 (G1): Group of patients with AI, a cortisol peak following LD-SST < 500 nmol/L or 18 µg/dL at T30 and T60: 39 patients (54.9%).
- Group 2 (G2): Group of healthy patients with a cortisol peak ≥ 500 nmol/L or 18 µg/dL at T30 or T60: 32 patients (45.1%).

The diagnostic threshold of 18 µg/dL (500 nmol/L) for defining adrenal insufficiency following Synacthen stimulation was selected based on its widespread acceptance in both pediatric and adult literature [1,2,6]. This cut-off has been validated across multiple assay platforms, including radioimmunoassay methods similar to those used in our study [6].

### 2.5. Statistical Analysis

After validation, the data were analyzed using SPSS software version 25. For comparisons, Student’s *t*-test was used for numerical variables, while chi-square tests were used for qualitative variables. The study of the association between two quantitative variables was carried out using the Pearson correlation coefficient.

To achieve good sensitivity and specificity in the LD-SST test, the search for the morning cortisol threshold was carried out using the Receiver-Operating Characteristic (ROC) curve. This allowed us to determine the optimal upper and lower threshold values for baseline cortisol and the cortisol increment for the diagnosis of IA.

## 3. Results

The mean age of the population was 6.23 ± 3.49 years with extremes ranging from 2 to 14 years. Male patients represented 57.7% (*n* = 41) of the study population. The sex ratio was 1.36. The etiologies justifying the child’s treatment with GCs were dominated by asthma for CSI and autoimmune diseases for oral corticosteroid therapy (18.3%), followed by nephrotic syndrome (8.4%), then bronchiolitis obliterans (7%) and epilepsy (4.2%). The mean duration of long-term corticosteroid therapy in our study population was 17.32 ± 13.54 months. For the inhaled form, the mean duration was 16.45 ± 13.54 months, compared with 18.74 ± 10.52 months for the oral form.

The symptoms assessed were mainly gastrointestinal disorders, including vomiting, bowel transit disturbances, and abdominal pain, observed in 14% of cases. For the clinical examination, the mean BMI was 16.80 ± 4.54 kg/m^2^ with extremes ranging from 9.72 kg/m^2^ to 34.72 kg/m^2^. Borderline high blood pressure was found in two patients. All the patients presented a normal pubertal stage according to the Tanner score. Biological disturbances were found in 70.42% of patients, such as anemia in 24 patients and hyponatremia in 11 children (Table 1).

### 3.1. Baseline Cortisol

The mean baseline cortisol level at T0 in our study population was 7.831 ± 2.55 µg/dL [Range: 2.53–15.05 µg/dL]. It was 6.69 ± 1.99 µg/dL for G1 [Range: 2.53–11.97 µg/dL]. It was significantly higher in G2 with 9.21 ± 2.49 µg/dL [Extremes: 5.99–15.05 µg/dL] (*p* < 10^−3^).

### 3.2. Cortisol After Synacthen

**Figure 1:**
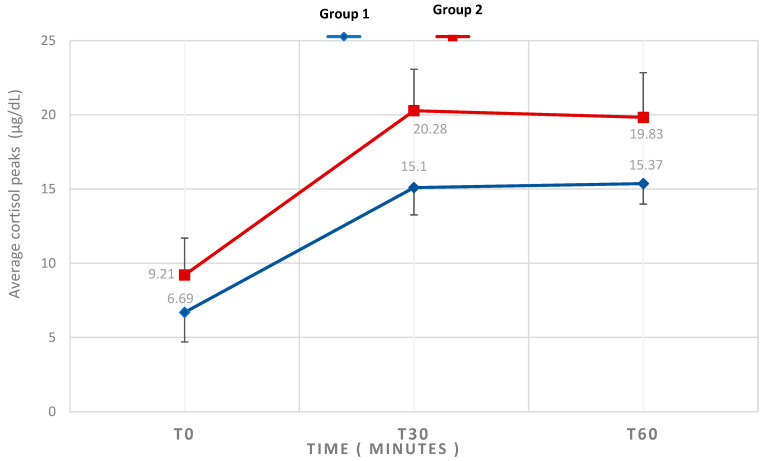
Mean cortisol peaks at LD-SST in both groups.

The mean serum cortisol values at T30 and T60 of the stimulation test were 17.29 ± 3.81 µg/dL and 17.38 ± 3.17 µg/dL, respectively (Table 2). The maximum cortisol response to the stimulation test in our population was obtained at T60 in 59.1% of cases and at T30 in 40.8% of cases (Figure 1).

For G2, cortisol reached a mean peak of 21.41 ± 2.15 µg/dL, ranging from 18.33 to 25.95 µg/dL, whereas for G1 the mean peak was 15.84 ± 1.50 µg/dL, ranging from 12.53 to 17.70 µg/dL.

The mean cortisol at T30 was 15.10 ± 1.84 µg/dL in G1, compared with 20.28 ± 2.79 µg/dL in G2. At T60, mean cortisol levels increased slightly in G1 with a level of 15.37 ± 1.38 µg/dL and decreased in G2 to 19.83 ± 3.01 µg/dL (Table 3). Indeed, the peak of cortisol was earlier in G1 compared to G2. At T30, cortisol levels peaked in 33.33% of G1 participants, compared to 53.12% in G2. At T60, the percentage of G1 participants reaching peak cortisol increased to 66.66%, whereas in G2, it decreased to 46.88%.

### 3.3. Cortisol Increment

The mean increase in cortisol was 10.54 ± 2.70 µg/dL, spanning a wide range from 5.28 to 17.97 µg/dL. At T30, the mean cortisol response was 9.61 ± 2.97 µg/dL, ranging from 0.01 to 17.97 µg/dL. At T60, the mean response was slightly lower at 9.55 ± 2.97 µg/dL, with values ranging from 1.89 to 15.43 µg/dL.

### 3.4. ACTH

The mean plasma ACTH concentration was 10.59 ± 5.44 pg/mL (2.33 ± 1.20 pmol/L) in G1 and 13.12 ± 8.72 pg/mL (2.89 ± 1.92 pmol/L) in G2. No significant difference was observed between the two groups (*p* = 0.541).

### 3.5. Post LD-SST Cut-Off Results

ROC curve analysis was performed to estimate the specificity and sensitivity of the test on baseline cortisol to predict the outcome of LD-SST. With an AUC of 0.823 (95% CI = 0.727–0.919), the diagnostic test has high accuracy (*p* < 10^−3^). The cortisol cut-off with the highest combined sensitivity and specificity was identified at 6 µg/dL, showing a sensitivity of 96.9%, a specificity of 46.2%, and a negative predictive value (NPV) of 92.8% (Table 4). Using a baseline cortisol level ≥6 µg/dL as a predictor of an adequate response to the LD-SST, 96.9% of cases (*n* = 68/71) were correctly categorized, with an NPV of 59.6%.

**Figure 2:**
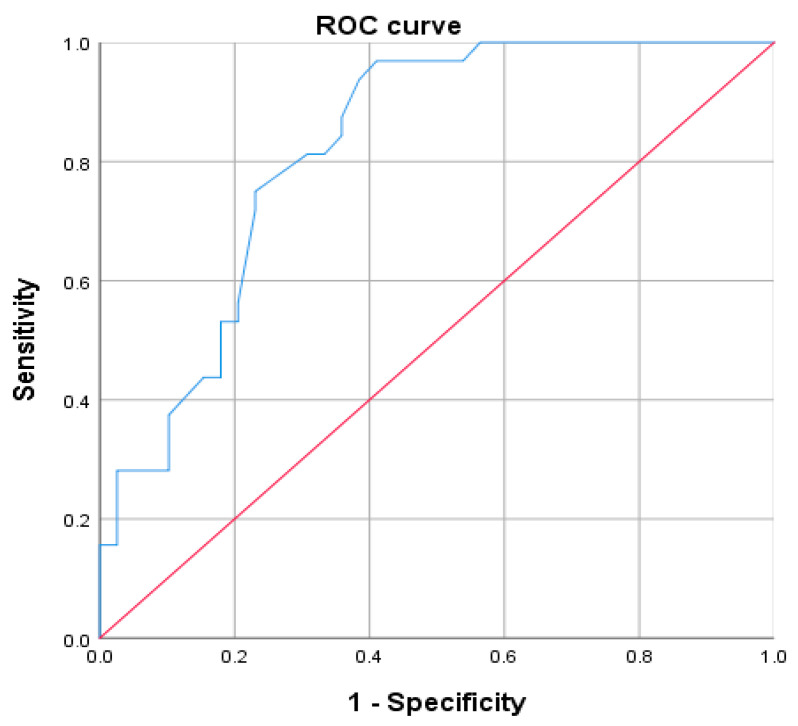
ROC curve and morning cortisol threshold during LD-SST. The y-coordinate expresses sensitivity and the x-coordinate expresses (1—specificity). The crossover point of a sensitivity of 96.9% and a specificity of 53.8% found a cortisol threshold = 6 μg/dL. The **blue line** represents the trade-off between sensitivity and specificity across various cortisol thresholds. The **red point** indicates the optimal cut-off point, corresponding to a morning cortisol threshold of 6 μg/dL, which yields a sensitivity of 96.9% and a specificity of 53.8%. The **diagonal dashed line** represents the line of no discrimination (or chance level), where the test performs no better than random chance.

ROC curve analysis was performed to estimate the specificity and sensitivity of the test on cortisol increment to predict the outcome of LD-SST. With an AUC of 0.822 (95% CI = 0.716–0.928), the diagnostic test has high accuracy (*p* < 10^−3^) (Figure 2). With 71 patients and an AUC of 0.823, our study had 89% power to detect a significant difference from the null hypothesis, which is adequate (>80%). However, for subgroup analyses and threshold-specific performance metrics (particularly the ≥13 µg/dL threshold where only 4 patients had cortisol above this level), the power is substantially lower, explaining the wide confidence intervals.

### 3.6. Subgroup Analyses

#### 3.6.1. Route of Corticosteroid Administration

Patients receiving oral corticosteroids had significantly higher odds of adrenal insufficiency compared to those on inhaled corticosteroids (OR = 5.06; 95% CI: 1.71–14.98; *p* = 0.002). Mean morning cortisol was significantly lower in the OCS group compared to the ICS group (6.94 ± 2.31 vs. 8.38 ± 2.57 μg/dL; *p* = 0.021).

#### 3.6.2. Treatment Duration

No significant difference in AI prevalence was observed based on treatment duration dichotomized at 12 months (*p* = 0.474). However, when analyzed as a continuous variable, longer treatment duration showed a weak positive correlation with AI (r = 0.23, *p* = 0.052).

#### 3.6.3. Underlying Condition

The highest AI prevalence was observed in autoimmune diseases (84.6%) and nephrotic syndrome (83.3%), conditions typically treated with systemic corticosteroids. Asthma, predominantly managed with ICS, had the lowest AI prevalence (40.9%) (Table 5).

## 4. Discussion

Prolonged or repeated corticosteroid therapy is a common medical practice, indicated for a wide range of pathologies [16]. However, data on optimal diagnostic strategies for glucocorticoid-induced adrenal insufficiency (GI AI) in pediatric populations remain limited, particularly in African settings. This pilot study represents an initial step toward addressing this gap.

Prolonged corticosteroid therapy remains a cornerstone of treatment for numerous pediatric conditions, yet its complication of glucocorticoid-induced adrenal insufficiency (GI-AI) poses significant diagnostic challenges. In our cohort of 71 children receiving long-term corticosteroids, the prevalence of AI was 54.9% as assessed by the low-dose Synacthen test. This finding aligns with the existing literature, which reports GI-AI in 28–100% of patients depending on the population studied and diagnostic criteria used [3,8]. Notably, among our patients receiving inhaled corticosteroids, 42.1% demonstrated AI, corroborating recent concerns raised by Zöllner et al. [15] and Hawcutt et al. [14] regarding the substantial risk of HPA axis suppression even with topical glucocorticoid administration. The higher prevalence observed in our oral corticosteroid subgroup (57.9%) was expected given the systemic exposure, though the overlap between groups underscores the unpredictable nature of individual susceptibility to adrenal suppression.

A key finding of our study was the significant association between morning basal cortisol levels and LD-SST outcomes. Patients with AI demonstrated markedly lower mean morning cortisol compared to those with normal adrenal function (6.69 ± 1.99 vs. 9.21 ± 2.49 μg/dL, *p* < 0.001). This association formed the rationale for exploring whether basal cortisol could serve as a predictive marker, potentially reducing reliance on dynamic testing. Notably, we found no correlation between basal ACTH levels and GI-AI (*p* = 0.541), consistent with a recent pediatric study [17], which similarly concluded that ACTH measurements lack sufficient diagnostic accuracy in this context. This observation likely reflects the pulsatile nature of ACTH secretion and its rapid degradation, making single measurements unreliable for assessing chronic HPA axis suppression.

ROC curve analysis demonstrated that morning cortisol predicts LD-SST response with good diagnostic accuracy (AUC = 0.823; 95% CI: 0.727–0.919). We identified two clinically useful thresholds: a lower cut-off of ≤6 μg/dL predicting AI with 96.9% sensitivity (95% CI: 83.8–99.9%), and an upper cut-off of ≥13 μg/dL predicting normal adrenal function with 100% specificity (95% CI: 89.1–100%). The cortisol increment following Synacthen stimulation also showed diagnostic value (AUC = 0.822), with an increment <9 μg/dL suggesting AI and ≥9 μg/dL effectively excluding the diagnosis. These findings suggest that incorporating both basal cortisol and post-stimulation increment into a diagnostic algorithm could improve diagnostic accuracy while reducing the false-positive rate associated with relying solely on a single T30 cortisol measurement [18,19,20,21,22].

Our identified thresholds demonstrate remarkable consistency with the limited pediatric data available. Maguire et al. [23] studied 31 children with suspected central adrenal insufficiency and reported that morning cortisol <5.07 μg/dL predicted AI while levels >10.47 μg/dL effectively excluded HPA axis dysfunction (96% sensitivity, 50% specificity). The slight differences in absolute values likely reflect variations in cortisol assays, population characteristics, and the reference standard used. Our upper threshold of 13 μg/dL, while higher than Maguire’s 10.47 μg/dL, provided perfect specificity in our cohort, though the wide confidence interval necessitates cautious interpretation. A 2008 meta-analysis by Kazlauskaite et al. [24] evaluating morning cortisol for assessing adrenal function reported optimal thresholds ranging from 3 to 15 μg/dL depending on the population and intended clinical use, further supporting the plausibility of our findings. More recently, Laulhé et al. [17] proposed that morning cortisol >12.5 μg/dL effectively excludes AI in children, closely approximating our 13 μg/dL threshold.

Based on these findings, we propose a structured diagnostic algorithm that could optimize resource utilization while maintaining diagnostic accuracy. Patients with basal cortisol ≤6 μg/dL could be diagnosed with AI without undergoing dynamic testing, while those with levels ≥13 μg/dL could be confidently reassured of normal adrenal function. For the approximately 70% of patients with indeterminate basal cortisol (6–13 μg/dL), LD-SST remains necessary. Following stimulation, a peak cortisol <18 μg/dL should prompt calculation of the cortisol increment, with values <9 μg/dL supporting the diagnosis of AI. Implementation of this algorithm in our cohort would have avoided LD-SST in 29.6% of patients, potentially reducing healthcare costs and patient burden. This approach aligns with recent calls for more efficient diagnostic strategies in pediatric endocrinology [17,25], particularly in resource-limited settings where dynamic testing may not be readily available.

Studies that test patients during active treatment [14,15] assess HPA function under real-world conditions but cannot distinguish drug effect from intrinsic dysfunction. Studies that use prolonged washout periods (4–12 weeks) [8] assess HPA function after allowing time for recovery but may underestimate the risk during the vulnerable post-withdrawal period. Our 7-day protocol assesses HPA function during the early withdrawal phase, which may be the most clinically relevant period for identifying patients at risk of adrenal crisis during intercurrent illness or stress, but it does not predict long-term recovery.

A cortisol increment of <9 µg/dL following stimulation suggests AI, whereas an increase of 9 µg/dL likely excludes the diagnosis, reducing the need for GC supplementation unless there is strong clinical suspicion.

Our study confirms that morning plasma cortisol concentrations are associated with response to LD-SST in children. This has been previously demonstrated for adult patients [26,27]. To our knowledge, only one pediatric study has assessed the association between baseline serum cortisol and LD-SST but did not assess thresholds to predict cortisol response to LD SST [16,26,27].

Considering the pediatric population, a retrospective study including 31 patients with suspected central AI assessed by a Synacthen test and 22 healthy controls showed that morning cortisol was associated with the test result. Thresholds for dynamic testing and 9 a.m. plasma cortisol levels were defined based on values obtained in healthy controls. Interestingly, the threshold value was 5.07 µg/dL. Furthermore, a morning plasma cortisol level above 10.47 µg/dL predicted a functional HPA axis with a sensitivity of 96% and a specificity of 50%, closely aligning with our findings [23].

We compared our findings with several studies, that evaluated the optimal lower and upper thresholds of morning plasma cortisol for assessing adrenal function, whose results also closely align with ours [24]. The concordance between our pilot data and this larger meta-analysis is encouraging, suggesting that our preliminary thresholds may be robust. However, given the age of this meta-analysis and the evolution of cortisol assays, updated pediatric-specific validation studies are urgently needed.

This study has several limitations that warrant consideration when interpreting our findings. Although our cohort of 71 patients provided 89% power to detect the primary ROC analysis (AUC = 0.823), the sample size is limited for establishing definitive diagnostic thresholds. This is particularly relevant for the upper threshold of ≥13 µg/dL, which was identified based on only 4 patients with cortisol levels above this value. Second, as a pilot study conducted at a single academic center in Tunisia, our findings may not be generalizable to other populations with different ethnic backgrounds, corticosteroid prescribing practices, or laboratory methods. Third, our cohort included children receiving both inhaled and oral corticosteroids for various indications. While this reflects real-world clinical practice, it introduces heterogeneity in terms of corticosteroid type, dosage, and duration of exposure, which may influence HPA axis suppression patterns.

## 5. Conclusions

This North African pilot study provides preliminary evidence that morning cortisol measurement may help predict LD-SST responses in children receiving long-term corticosteroid therapy. We propose a diagnostic algorithm using a lower threshold of ≤6 µg/dL (suggesting adrenal insufficiency) and an upper threshold of ≥13 µg/dL (suggesting normal adrenal function), which could potentially reduce unnecessary dynamic testing by approximately 30%. Large, multicenter prospective studies are needed to validate these thresholds across diverse pediatric populations, evaluate their cost-effectiveness, and determine their generalizability to different cortisol assays and healthcare settings. Until such validation is available, clinical judgment should guide the use of morning cortisol in combination with dynamic testing. The 7-day washout period used in this study, while sufficient for drug clearance, is inadequate for complete HPA axis recovery, which may require weeks to months. Therefore, our findings reflect the state of HPA suppression during the early withdrawal phase, not permanent adrenal insufficiency. Patients with cortisol ≤6 µg/dL at 7 days require repeat testing after longer intervals (4–12 weeks) to distinguish transient suppression from persistent insufficiency requiring long-term glucocorticoid replacement.

## Figures and Tables

**Table 1:** Clinical, biological, and therapeutic characteristics of the study population.

	Population (n)	Percentage (%)
**Etiologies (*n*):**		
Asthma	44	61.9
Nephrotic syndrome	6	8.4
Autoimmune diseases	13	18.3
Epilepsy	3	4.2
Bronchiolitis obliterans	5	7
**Corticosteroid therapy:**		
Age of start of treatment (years)	6.23 ± 3.49	
Processing time (months)	17.32 ± 13.4	
**Method of administration (*n*)**		
Inhaled	44	61.9
Oral	27	38
**Molecule (*n*)**		
Prednisone	7	9.8
Prednisolone	20	28.1
Fluticasone propionate	27	38
Budesonide	6	8.4
Beclomethasone	8	11.2
**Clinical data**		
BMI (kg/m)	16.80 ± 4.54 [9.72–34.72]	-
High blood pressure (*n*)	2	2.8
Pubertal abnormalities (*n*)	0	0
Digestive disorders (diarrhea, vomiting, etc.) (*n*)	10	14
Sleep disorder, headache (*n*)	1	1.4
**Biological Data (*n*)**		
Anemia	24	33.8
Hypocalcemia	3	4.2
Hyponatremia	11	15.4
Hyperphosphoremia	9	12.6
Hyperglycaemia	1	1.4

BMI: Body mass index.

**Table 2:** Distribution of cortisol levels and frequency of maximum responses at different sampling times during LD-SST in the study population.

Time (Minutes)	T0	T30	T60
Mean cortisol (μg/dL)	7.83 ± 2.55	17.29 ± 3.81	17.38 ± 3.17
Patients achieving peak cortisol, *n* (%)	NA	40.8	59.1

T0: Time 0 min; T30: Time 30 min; T60: Time 60 min.

**Table 3:** Summary table of distribution of cortisol levels and frequency of peaks at different time points during LD-SST in the two study groups.

Time (Minutes)		T0	T30	T60
**Average cortisol (μg/dL)**	**G1**	6.69 ± 1.99	15.10 ± 1.84	15.37 ± 1.38
	**G2**	9.21 ± 2.49	20.28 ± 2.79	19.83 ± 3.01
**Patients achieving peak cortisol, *n* (%)**	**G1**	NA	13	26
	**G2**	NA	17	15

G1: Group 1; G2: Group 2; T0: Time 0 min; T30: Time 30 min; T60: Time 60 min.

**Table 4:** Diagnostic performance of baseline cortisol measurements after LD-SST.

Cortisol Threshold (µg/dL)	True Positives (TP)	False Negatives (FN)	True Negatives (TN)	False Positives (FP)	Sensitivity (%) [95% CI]	Specificity (%) [95% CI]	PPV (%) [95% CI]	NPV (%) [95% CI]
3.7	39	0	1	31	100 [89.1–100]	3.1 [0.08–16.2]	55.7 [54.2–57.2]	100 [NA]
6	38	1	18	14	97.4 [86.5–99.9]	56.3 [37.7–73.6]	73.1 [62.5–81.6]	94.7 [71.9–99.3]
13	4	35	32	0	10.3 [2.9–24.2]	100 [89.1–100]	100 [NA]	47.8 [45.6–50.0]

TP: True Positive (AI correctly identified by threshold), FN: False Negative (AI missed by threshold), TN: True Negative (normal correctly identified), FP: False Positive (normal misclassified as AI). AI was defined as peak cortisol <18 µg/dL on LD-SST (*n* = 39). In this table, two distinct thresholds are presented with different clinical purposes. The ≤6 µg/dL threshold is intended to diagnose adrenal insufficiency (rule-in). At this threshold, 38 of 39 patients with AI (97.4%) had baseline cortisol ≤6 µg/dL (true positives), while 14 of 32 patients with normal adrenal function (43.7%) also had cortisol ≤6 µg/dL (false positives), yielding a specificity of 56.3%. Conversely, the ≥13 µg/dL threshold is intended to exclude adrenal insufficiency (rule-out). Only 4 patients in our cohort had baseline cortisol ≥13 µg/dL, all of whom had normal adrenal function on LD-SST. Thus, a cortisol level ≥13 µg/dL was 100% specific for normal adrenal function, though the sensitivity for detecting normal patients was only 12.5% due to the small number of patients reaching this threshold. The negative predictive value for AI (i.e., probability of having normal adrenal function if cortisol ≥13 µg/dL) was 100%, though this estimate is unstable due to small sample size (*n* = 4).

**Table 5:** AI frequency based on underlying condition.

Condition	*N*	AI (*n*, %)	Normal (*n*, %)
Asthma	44	18 (40.9%)	26 (59.1%)
Autoimmune diseases	13	11 (84.6%)	2 (15.4%)
Nephrotic syndrome	6	5 (83.3%)	1 (16.7%)
Bronchiolitis obliterans	5	3 (60.0%)	2 (40.0%)
Epilepsy	3	2 (66.7%)	1 (33.3%)

## Data Availability

The original contributions presented in this study are included in the article. Further inquiries can be directed to the corresponding author.
